# The Laws of Epidemics

**Published:** 1856-10

**Authors:** 


### THE LAWS OF EPIDMIS.

Dr. Bell Salter, at the twenty-fourth anniversary meeting of the Provincial Medical and Surgical Association, read the address in medicine, which was a summary of our present knowledge of the laws of epidmis. He said that the lower animals and plants were subject to epidemic diseases, some of which had been found consentaneous with others prevailing amongst mankind. Wild birds were often known to be scarce or absent from places where human epidemics prevailed; and their return was a sign that the intensity of the disease had diminished. Flies are often few at periods of epidemics, though at other times the presence of large numbers of insects is coincident with the disease; and the multitude of some, as locusts, is an active cause of certain maladies. The speaker proceeded to classify epidemic diseases, and indicated the years of some of their most serious attacks. Of the older epidemics, the plague and the yellow fever were the principal ones remaining; but the former, for the most part, ever since the fearful plague of London, in 1665, had been confined to parts of Asia and Africa, bordering the Mediterranean. Amongst epidemics mentioned in history, but now extinct, (?) is the sweating sickness, which, in some cases, had been known to carry off the patient in two hours: it first appeared in Europe, 1484; then prevailed from 1517 to 1528; and was last known in England in 1550; but in Denmark and Sweden, it had prevailed so late as 1710. The black-death, which first appeared in 1348, had, like cholera, arisen in the east, and spread westward: it appeared to have been a modification of plague in conjunction with pneumonia. Leprosy and cholera were epidemics of the thirteenth and fourteenth centuries; and the latter (like some manifestations which have within the last few years been observed in Germany and Sweden) had apparently a mental origin. Irregularity of the seasons had always attended the outbreak of an epidemic; and an extreme paucity of house flies had been noticed during the attacks of cholera in 1832 and 1849. The history of an epidemic outbreak was next described; and it was remarked that the localities in which the first cases had appeared were those where the heaviest amount of mortality had been present to the last. The mortality from modern epidemics is, however, trifling, as compared with that caused by the old ones. Of those attacks by the sweating sickness, only one or two per cent. were accustomed to survive. Epidemics were then classified into local, latitudinal, and mundane. Amongst many other subjects, Dr. Bell Salter drew attention to the report of the Board of Health on cholera, and the proofs furnished by it that the production of that disease was intimately connected with impurities in the water supply; that little electricity had been manifested, and a total absence of ozone from the atmosphere during attacks of cholera; that the zymotic doctrine is more applicable to cholera than has been yet recognised; that epidemics are not affected by regulations of quarantine; and that intervals free from severe epidemics were the proper periods for making and carrying out arrangements to prevent them, which in times of pestilence are fraught with danger. He remarked that tontines have proved the value of human life to be one-fourth greater than in the century before the time of Pitt; that medical science had done much to contribute to this result; and that the more we were enabled to check the progress of minor epidemics, the less was the liability to others of a more severe description.—*London Lancet*

